# Sex estimation accuracy through metric evaluation of dental root: a cone-beam computed tomography-based study

**DOI:** 10.1590/0103-644020256063

**Published:** 2025-04-07

**Authors:** Daniele Manhaes-Caldas, Matheus L Oliveira, Francisco Carlos Groppo, Dagmar de Paula Queluz

**Affiliations:** 1 Department of Oral Diagnosis, Division of Oral Radiology, Piracicaba Dental School, University of Campinas, Piracicaba, São Paulo, Brazil.; 2 Department of Bioscience, Division of Pharmacology, Anesthesiology and Therapeutic Drugs, Piracicaba Dental School, University of Campinas, Brazil.; 3 Department of Health Sciences and Children’s Dentistry, Piracicaba Dental School, University of Campinas, Brazil.

**Keywords:** cone-beam computed tomography, tooth root, sexual dimorphism

## Abstract

The degree of sexual dimorphism exhibited by teeth plays a crucial role in sex estimation, particularly as they are frequently found well-preserved. This study aimed to assess the presence of sexual dimorphism in the roots of upper lateral incisors, upper canines, and lower canines using cone-beam computed tomography (CBCT), and to develop and validate a formula for sex estimation. Linear measurements, surface area, and volumetric measurements were conducted on a total of 140 CBCT volumes (100 for evaluation and 40 for validation) from a Brazilian population, with an equal distribution of sexes, aged between 18 and 50. Statistical analysis involved the Kolmogorov-Smirnov test, unpaired t-test with Welch correction, Pearson's correlation test, logistic regression using the Backward Stepwise method, sieve test, Kappa test, intraclass correlation coefficient, and statistical power calculation (α=0.05). The three teeth showed sexual dimorphism, with measurements from males being statistically larger. Notably, only the formulas for the lower canine met the Mohan and Daubert admissibility criteria. The formula for assessing a sound root used included the mesiodistal diameter, axial cross-section volume, and two-thirds of the coronal cross-section volume. For evaluating reduced root length, the formula incorporated the mesiodistal diameter and axial cross-section volume. The accuracy of the lower canine formulas for sound and reduced root length were 89% and 87%, respectively. After validation, both formulas achieved an accuracy of 85%. In conclusion, the models combining measurements from the lower canine can be applied as a complementary method for sex estimation.

## Resumo

O grau de dimorfismo sexual exibido pelos dentes desempenha um papel crucial na estimativa de sexo, especialmente considerando que eles são frequentemente encontrados bem preservados. O objetivo deste estudo foi avaliar a presença de dimorfismo sexual nas raízes dos incisivos laterais superiores, caninos superiores e caninos inferiores utilizando tomografia computadorizada de feixe cônico (TCFC), e desenvolver e validar uma fórmula para a estimativa de sexo. Foram realizadas medições lineares, de área superficial e volumétricas em um total de 140 volumes de TCFC (100 para avaliação e 40 para validação) de uma população brasileira, com distribuição igual entre os sexos, com idades entre 18 e 50 anos. A análise estatística envolveu o teste de Kolmogorov-Smirnov, o teste t não pareado com correção de Welch, o teste de correlação de Pearson, regressão logística utilizando o método Backward Stepwise, teste de peneira, teste Kappa, coeficiente de correlação intraclasse e cálculo do poder estatístico (α=0,05). Os três dentes apresentaram dimorfismo sexual, com as medições dos homens sendo estatisticamente maiores. Notavelmente, apenas as fórmulas para o canino inferior atenderam aos critérios de admissibilidade de Mohan e Daubert. A fórmula para a avaliação de uma raiz hígida incluiu o diâmetro mesiodistal, o volume da seção transversal axial e dois terços do volume da seção transversal coronária. Para a avaliação do comprimento reduzido da raiz, a fórmula incorporou o diâmetro mesiodistal e o volume da seção transversal axial. A precisão das fórmulas para o canino inferior para comprimento de raiz hígida e reduzida foram 89% e 87%, respectivamente. Após a validação, ambas as fórmulas alcançaram uma precisão de 85%. Em conclusão, os modelos que combinam medições do canino inferior podem ser aplicados como um método complementar para a estimativa de sexo.

**Figure:**
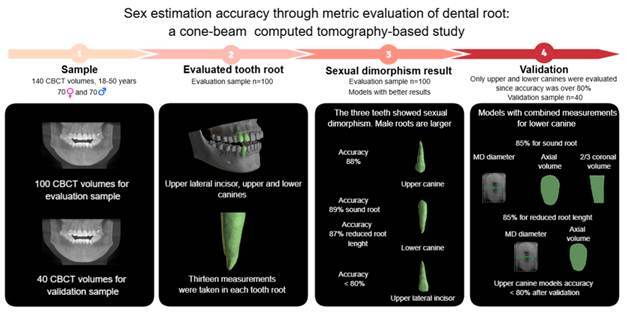

## Introduction

The establishment of biological identity depends, among other steps, on the sex estimation. The importance is also observed because its establishment simplifies the identification process, as only missing persons of the estimated sex will be considered ^1^. The deoxyribonucleic acid (DNA) analysis is considered the reference standard in the forensic context. However, it is expensive and becomes unfeasible if the biological material is degraded ^2^. When this occurs, forensic anthropologists analyze body structures that exhibit sexual dimorphism. The pelvis and postcranial bones are the most dimorphic structures. In their absence, the skull represents a valuable tool ^3^. However, these structures are commonly missing or even partially damaged, making their evaluation unfeasible.

In this context, teeth have demonstrated a promising role in estimating sex since, contrary to what occurs with bone, they do not undergo remodeling. Thus, it represents a more stable structure ^4^. In addition, they are often recovered with their structure intact ^2^, as they are highly resistant to mechanical, chemical, physical, and thermal effects ^5^. They also count on the protection provided by orofacial tissues ^2^. Thus, numerous studies have evaluated teeth' metric characteristics (5-8). These studies identified significant tooth sexual dimorphism, with male teeth typically being larger than female teeth ^2^ ^,^ ^6^ ^,^ ^9^.

Greater focus has been placed on the metric analysis of dental crowns. However, studies comparing linear measurements of the root cervical region and the dental crown have indicated that cervical measurements as more dimorphic ^10^ ^,^ ^11^ ^,^ ^12^. In addition, dental roots offer a better alternative as they are less affected by pathologies and wear ^13^. Although sexual dimorphism has been identified in all tooth types, upper and lower canines are commonly recognized as the most dimorphic among single-rooted teeth ^11^. However, specific evaluations of the root portion have suggested that the root of the upper lateral incisors exhibits even greater dimorphism than that of the canines ^13^ ^,^ ^14^. It should be noted that, while isolated measurements show dimorphism, combining assessments of different tooth types ^6^ ^,^ ^10^ ^,^ ^11^ ^,^ ^14^ and measurements ^5^ ^,^ ^11^, as well as integrating teeth with other structures such as the mandible ^15^, can enhance the accuracy of sex estimation. Moreover, recent studies employing artificial intelligence for metric analysis in sex estimation have also reported promising outcomes ^8^ ^,^ ^15^.

With the increased accessibility of micro-tomography, cone-beam computed tomography (CBCT), and multidetector computed tomography (MDCT), along with their widespread adoption in numerous forensic institutes ^16^, new methodologies have been developed to improve the accuracy of sex estimation. The acquisition of CT images has further facilitated their application in forensic odontology, where dental radiology plays a fundamental role ^16^.

According to Zhang ^16^, the use of CT offers new possibilities for comparing *antemortem and postmortem* dental information in the identification process, as well as its application in age estimation ^16^. In the context of sex estimation, CT images enable the evaluation of previously inaccessible dental measurements, such as volumetric evaluation of the root ^14^ ^,^ ^17^ and its surface area ^4^, thereby enhancing accuracy. Additionally, CT images allow for evaluations without magnification, geometric distortions, or overlaps ^5^, making measurements comparable to direct observations ^18^. Importantly, the accuracy of CBCT and MDCT measurements has been shown to be equivalent ^19^.

Another advantage of CT images is their ability to analyze fragile structures without the need for physical manipulation, thus preventing the loss of structural integrity that could compromise evaluation results ^6^. Consequently, the present study aimed to investigate sexual dimorphism in the roots of upper lateral incisors, upper canines, and lower canines using linear, surface area, and volumetric measurements derived from CBCT images. Additionally, the study sought to develop and validate a formula for sex estimation. The null hypothesis was that the examined teeth did not exhibit sexual dimorphism.

## Materials and methods

### Sample

**Figure 1:**
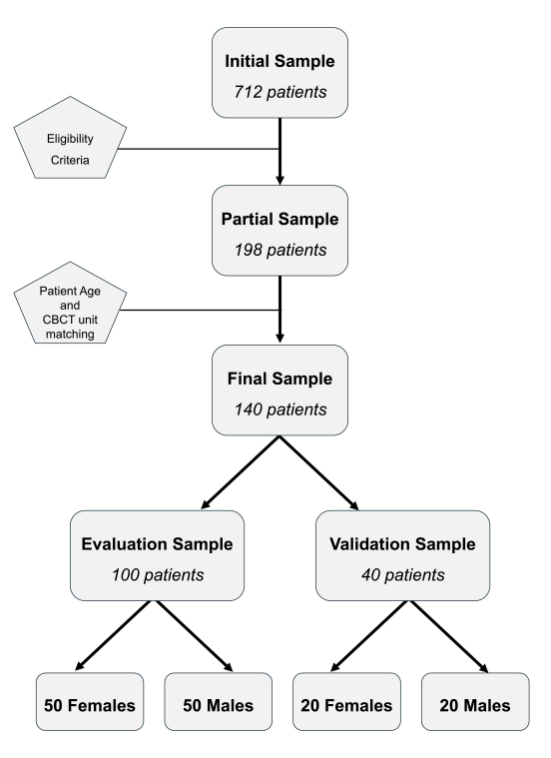
Flowchart detailing the process of determining the sample size, from the initial patient sample to the evaluation and validation subsets.

This study was approved by the local institution's research ethics committee (protocol CAAE 46375121.6.0000.5418). A total of 712 patients had their CBCT images evaluated and were subjected to eligibility criteria. The inclusion criteria required the presence of the following three specific teeth with fully formed roots in each patient: one upper lateral incisor, one upper canine, and one lower canine. Measurements were primarily taken on the left side; in instances of exclusion criteria, the contralateral side was used ^1^ ^,^ ^4^ ^,^ ^7^ ^,^ ^10^. The exclusion criteria were the presence of motion artifacts that resulted in a double contour of the structures in the analyzed region, orthodontic appliances, and orthodontic retainers on the lingual surface of the lower anterior teeth (which may suggest previous orthodontic treatment), root fractures, carious lesions, wear, restorations involving the cervical root region, endodontic treatment, periapical lesions - even with intact dental crowns -indicating potential prior dental trauma, root resorption, including apical rounding, which may indicate prior orthodontic treatment, root dilaceration, and any factor related to size, number, or development of the tooth root. After applying these criteria, a partial sample of 198 patients was obtained. Further exclusions were due to the lack of patients of the opposite sex for matching age and CBCT equipment. Consequently, the final sample consisted of 140 patients with equal sex distribution (70 females and 70 males) aged between 18 and 50 from a Brazilian population. This sample was randomly divided into two subsets: 100 patients (50 females and 50 males) with matched ages formed the evaluation sample used to obtain the sex estimation formula, while the remaining 40 patients (20 females and 20 males) constituted the validation sample (Figure 1).

The CBCT volumes were retrieved from the image archive of the dental school's radiology clinic. They were acquired using two different devices for clinical purposes unrelated to this study, all within the same institution: ^1^ Picasso Trio (Vatech, Hwaseong, Republic of Korea) with the following acquisition parameters: 80 kVp, 3.7 mA, 0.2 mm voxel size and ^2^ i-CAT (Imaging Sciences International, Hatfield, PA, USA), with the following acquisition parameters: 120 kVp, 3.6-4.8 mA, 0.25-0.4 mm voxel size. The voxel was resized to 0.12 mm using the open-source 3D Slicer software, version 4.8.1 ^20^, to ensure the study’s methodology could be applied across images, irrespective of the original voxel size.

### CBCT-based Measurements

**Figure 2:**
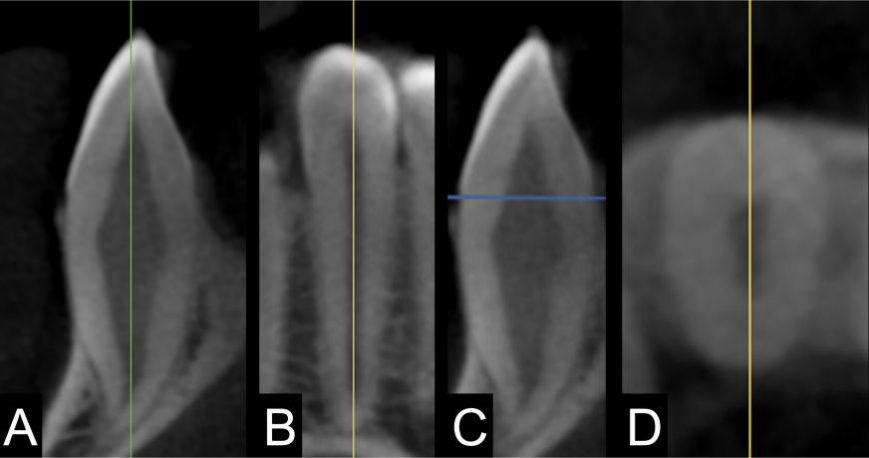
Representative cone-beam computed tomography cross sections: (A) sagittal and (B) coronal views highlighting the alignment of the CBCT volume with the longitudinal axis of the tooth; (C) sagittal view highlighting the determination of the cervical region and (D) the resulting axial view.

An experienced observer in CBCT and tooth segmentation performed o total of 13 measurements in each tooth. Given the varying positions of teeth within the dental arches, the CBCT volumes were spatially reoriented for each tooth under assessment by aligning the longitudinal axis of the tooth with the sagittal (Figure 2A) and coronal planes (Figure 2B) to obtain appropriate buccolingual and mesiodistal perspectives, as recommended by Kim et al.^18^. Furthermore, the cervical level was established as an imaginary plane intersecting the most apical portions of the enamel on both the buccal and palatal/lingual surfaces, as suggested by Ma et al. ^21^ (Figure 2C, 2D).

**Figure 3:**
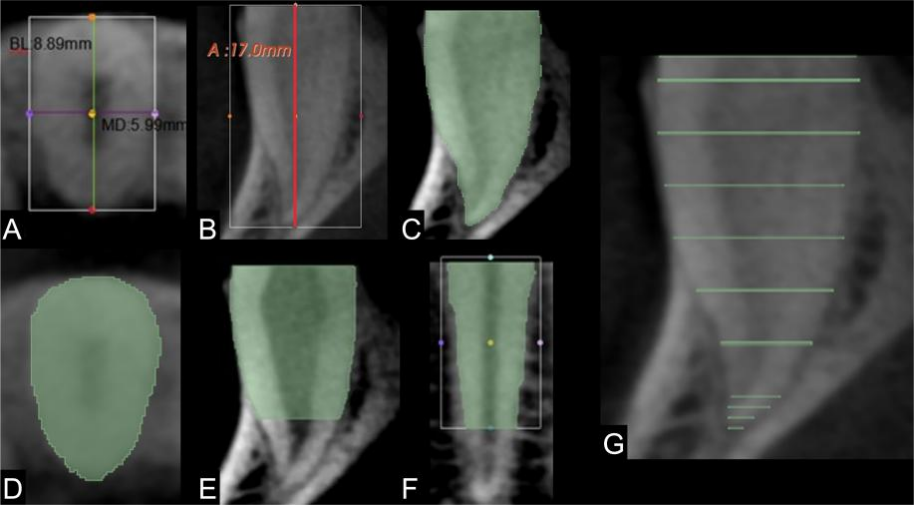
Representative CBCT cross-sections illustrating the tooth measurements: (A) buccolingual and mesiodistal cervical diameters; (B) root length; (C) surface area and volume of the root; (D) axial surface area and volume at the cervical level; (E) sagittal surface area and volume of the cervical two-thirds of the root; (F) coronal surface area and volume of the cervical two-thirds of the root; (G) axial intervals for image segmentation.

 The 13 measurements were as follows: 1) buccolingual cervical diameter (1a); 2) mesiodistal cervical diameter (1b); 3) root length (1c); 4) surface area of the root (2), 5) volume of the root (3); 6) surface area of the cervical two-thirds of the root (4); 7) volume of the cervical two-thirds of the root (5); 8) axial surface area at the cervical level (6a); 9) sagittal surface area at the longitudinal axis of the cervical two-thirds of the root (6b); 10) coronal surface area at the longitudinal axis of the cervical two-thirds of the root (6c), 11) axial volume at the cervical level (7a), 12) sagittal volume at the longitudinal axis of the cervical two-thirds of the root (7b); 13) coronal volume at the longitudinal axis of the cervical two-thirds of the root (7c). (Figure 3A-F).

To obtain measurements 4 through 8, and 11, the root was manually segmented from axial reconstructions. The apical third of the root was segmented at intervals of four axial cross-sections, while the cervical two-thirds were segmented at intervals of twenty axial cross-sections (Figure 3G). This approach was employed because the apical region exhibits the most significant changes in shape and angulation. Subsequently, the “fill between slices” tool was used to complete the root segmentation, after which the surface area (in mm²) and volume (in mm³) were obtained. To obtain measurements 9 (6b) and 12 (7b), the central sagittal reconstruction was manually segmented. To obtain measurements 10 (6c) and 13 (7c), the central coronal reconstruction was manually segmented. To estimate the intra-examiner reliability, 20% of the sample was reassessed after 30 days.

### Statistical analysis

IBM SPSS Statistics version 25 and GraphPad Prism version 8 (GraphPad Software, La Jolla, CA, USA) were used for statistical analysis. Intra-examiner reliability was assessed using the intraclass correlation coefficient (ICC). The distribution of isolated measurements was verified using the Kolmogorov-Smirnov test, while differences between sexes were compared using the unpaired t-test with Welch’s correction. Pearson’s correlation test was applied to assess the relationship between age and tooth measurements. The predictive model formulas were generated using logistic regression via the Backward Stepwise method, both with individual measurements and in combination. The sieve test was used to determine the sensitivity, specificity, and accuracy of these models. The Kappa test was performed to validate the proposed models, indicating the level of agreement between observed outcomes and those predicted by the formula, and interpreted according to Landis and Koch ^(22)^. A significance level of 5% was applied to all tests. A sample size of 50 patients per sex was sufficient to achieve a test power of 95%, assuming a 5% significance level for a two-tailed, unpaired test (α=0.05).

## Results

The intraobserver correlation was excellent, with an ICC > 0.9. The calculation of intra-examiner agreement was performed only for those measurements included in the equations with the highest accuracy in sex estimation (upper canine measurements 1b, 1c, 3, 7a, and 7b; lower canine measurements 1b, 1c, 7a, and 7c) (Table S1). The unpaired t-test with Welch correction showed that, when analyzed separately by tooth type, there was a statistically significant difference between sexes, with measurements being significantly higher in men, regardless of the tooth type (Table 1). 

**Table 1:** Mean values and standard deviations of all measurements performed on the upper lateral incisor and upper and lower canines.

	Upper lateral Incisor			Upper Canine			Lower Canine		
M	Female	Male	p	Female	Male	p	Female	Male	p
1a	6.01 ±0.6 (5.84 - 6.18)	6.36 ±0.46 (6.23 - 6.49)	0.0014	7.69 ±0.63 (7.51 - 7.87)	8.49 ±0.69 (8.29 - 8.68)	<0.0001	7.19 ±0.57 (7.03 - 7.36)	8.05 ±0.63 (7.87 - 8.23)	0.0251
1b	4.53 ±0.42 (4.41 - 4.65)	4.93 ±0.54 (4.78 - 5.09)	<0.0001	5.38 ±0.34 (5.29 - 5.48)	6.01 ±0.45 (5.88 - 6.13)	<0.0001	4.95 ±0.29 (4.87 - 5.04)	5.56 ±0.38 (5.45 - 5.67)	0.0003
1c	12.86 ±1.65 (12.39 - 13.33)	13.6 ±1.58 (13.15 - 14.05)	<0.0001	16.72 ±1.95 (16.17 - 17.28)	18.24 ±2.12 (17.64 - 18.84)	<0.0001	15.06 ±1.41 (14.66 - 15.46)	16.43 ±1.59 (15.98 - 16.88)	<0.0001
2	193.6 ±31.5 (184.6 - 202.6)	219.3 ±29.61 (210.9 - 227.8)	<0.0001	295.6 ±42.24 (283.6 - 307.6)	360.4 ±48.82 (346.5 - 374.2)	<0.0001	265.7 ±33.69 (256.1 - 275.2)	319.5 ±39.3 (308.3 - 330.7)	<0.0001
3	173.1 ±36.39 (162.8 - 183.5)	209 ±40.21 (197.5 - 220.4)	<0.0001	313.6 ±57.97 (297.1 - 330)	426.2 ±76.79 (404.4 - 448)	<0.0001	272.5 ±47.67 (259 - 286.1)	362.1 ±63.66 (344 - 380.2)	<0.0001
4	163.6 ±25.72 (156.3 - 171)	185.7 ±25.6 (178.5 - 193)	<0.0001	252.5 ±34.99 (242.5 - 262.4)	309.3 ±41.54 (297.5 - 321.1)	<0.0001	227.3 ±27.18 (219.5 - 235)	275.8 ±34.69 (265.9 - 285.6)	<0.0001
5	148.3 ±31.04 (139.5 - 157.1)	179.5 ±35.38 (169.4 - 189.5)	<0.0001	275.7 ±50.5 (261.4 - 290.1)	376.4 ±69.01 (356.8 - 396)	<0.0001	236.7 ±39.28 (225.6 - 247.9)	317.8 ±57.22 (301.5 - 334.1)	<0.0001
6a	41.68 ±6.59 (39.81 - 43.55)	48.14 ±7.8 (45.92 - 50.36)	<0.0001	63.32 ±7.5 (61.19 - 65.45)	76.6 ±9.21 (73.98 - 79.22)	<0.0001	54.99 ±6.35 (53.18 - 56.8)	69.92 ±9.22 (67.3 - 72.54)	<0.0001
6b	86.24 ±16.81 (81.46 - 91.02)	96.87 ±14.86 (92.65 - 101.1)	0.0011	142.7 ±24.83 (135.7 - 149.8)	177.5 ±31.75 (168.4 - 186.5)	<0.0001	127.6 ±20.06 (121.9 - 133.3)	156.6 ±22.75 (150.2 - 163.1)	<0.0001
6c	59.22 ±11.09 (56.07 - 62.37)	67.5 ±12.11 (64.05 - 70.94)	0.0006	88.57 ±13.84 (84.64 - 92.51)	107 ±15.26 (102.6 - 111.3)	<0.0001	74.4 ±10.74 (71.35 - 77.46)	92.7 ±12.63 (89.11 - 96.29)	<0.0001
7a	2.4 ±0.39 (2.29 - 2.51)	2.99 ±0.95 (2.72 - 3.26)	<0.0001	3.67 ±0.44 (3.55 - 3.8)	4.46 ±0.54 (4.3 - 4.61)	<0.0001	3.18 ±0.37 (3.07 - 3.29)	4.14 ±0.77 (3.92 - 4.36)	<0.0001
7b	5.01 ±0.99 (4.73 - 5.3)	5.64 ±0.88 (5.39 - 5.89)	0.0012	8.36 ±1.47 (7.94 - 8.78)	10.51 ±1.91 (9.97 - 11.05)	<0.0001	7.47 ±1.19 (7.13 - 7.81)	9.19 ±1.35 (8.81 - 9.58)	<0.0001
7c	3.4 ±0.65 (3.22 - 3.59)	3.89 ±0.71 (3.69 - 4.1)	0.0005	5.13 ±0.82 (4.9 - 5.36)	6.2 ±0.96 (5.93 - 6.47)	<0.0001	4.3 ±0.63 (4.12 - 4.48)	5.38 ±0.75 (5.17 - 5.59)	<0.0001

M, measurements; 1a, buccolingual cervical diameter; 1b, mesiodistal cervical diameter; 1c, root length; 2, surface area of the root; 3, the volume of the root; 4, the surface area of the cervical two-thirds of the root; 5, the volume of the cervical two-thirds of the root; 6a, axial surface area at the cervical level; 6b, sagittal surface area at the longitudinal axis of the cervical two-thirds of the root; 6c, coronal surface area at the longitudinal axis of the cervical two-thirds of the root; 7a, axial volume at the cervical level, 7b, sagittal volume at the longitudinal axis of the cervical two-thirds of the root; 7c, coronal volume at the longitudinal axis of the cervical two-thirds of the root.

Pearson's correlation coefficient (rP) showed that there was no significant correlation between age and measurements for any of the teeth (rP < 0.2, p > 0.05). The model that evaluated measurements and tooth types separately showed that no measurement of the upper lateral incisor achieved accuracy greater than 80% (Table 2). Concerning the upper canine, only measurements 6a and 7a showed an accuracy greater than 80%. Both models achieved an overall accuracy of 82%, with a cut-off value of 0.5 (values greater than 0.5 indicating male) (Table 2). Regarding the lower canine, only the models incorporating measurements 3, 4, 5, 6a, and 7a exhibited accuracy exceeding 80% for each sex (Table 2).

**Table 2:** Accuracy values (in percentage) in sex estimation when analyzing and measuring each tooth separately.

Measurement	Upper Lateral Incisor	Upper Canine	Lower Canine
1a	66	70	78
1b	62	80 (F=82, M=78)	80 (F=82, M=78)
1c	57	66	69
2	65	76	79
3	69	82(F=86, M=78)	82(F=82, M=82)
4	67	79	81(F=82, M=80)
5	68	81(F=84, M=78)	82(F=82, M=82)
6a	68	82(F=84, M=80)	84(F=82, M=86)
6b	66	77	77
6c	68	76	75
7a	69	82(F=84, M=80)	84(F=82, M=86)
7b	66	78	77
7c	68	77	75

1a, buccolingual cervical diameter; 1b, mesiodistal cervical diameter; 1c, root length; 2, surface area of the root; 3, volume of the root; 4, surface area of the cervical two-thirds of the root; 5, volume of the cervical two-thirds of the root; 6a, axial surface area at the cervical level; 6b, sagittal surface area at the longitudinal axis of the cervical two-thirds of the root; 6c, coronal surface area at the longitudinal axis of the cervical two-thirds of the root; 7a, axial volume at the cervical level, 7b, sagittal volume at the longitudinal axis of the cervical two-thirds of the root; 7c, coronal volume at the longitudinal axis of the cervical two-thirds of the root. The underlined values are the only ones where both overall and sex-specific accuracies meet or exceed 80%.

For the upper lateral incisor, the model that obtained the highest accuracy and was statistically significant (Chi-square, p<0.0001) combined the predictive measurements 1c and 5. This model showed a sensitivity of 66%, specificity of 76%, and an overall accuracy of 71% (female = 76%, male = 66%), with a cut-off value of 0.5 (values greater than 0.5 indicating male). The model incorporating measurements 1c and 5 was also applied to the upper and lower canines to assess whether a universal formula could be used across all teeth. However, this model did not yield the highest accuracy for these teeth. For the upper canine, the model provided an overall accuracy of 84% (female = 90%, male = 78%), while for the lower canine, the overall accuracy was 85% (female = 86%, male = 84%). 

When analyzing the model considering only the upper canine, the most effective measurements were 1b, 1c, and 3. This model achieved an overall accuracy of 88% (female = 92%, male = 84%), with a sensitivity of 84% and a specificity of 92%. However, the validation accuracy for this model, despite a reasonable agreement (kappa = 0.25, p = 0.055), was only 63%. This same model was also evaluated for the upper lateral incisor (total accuracy = 71%) and the lower canine (total accuracy = 88%). Nonetheless, this model did not outperform the specific models developed for each tooth.

The model for the lower canine that achieved the highest accuracy was derived using measurements 1b, 7a, and 7c (Table 3), resulting in an overall accuracy of 89% (female = 88%, male = 90%), with a sensitivity of 89.8% and a specificity of 88.2%. The validation accuracy of the formula (sex = -31.69+(2.781*1b) +(2.094*7a) +(2.02*7c)), based on these measurements, demonstrated substantial agreement (Kappa = 0.7, p < 0.0001) with an overall accuracy of 85% (female = 85%, male = 85%). When applied to the upper lateral incisor (total accuracy = 72%) and upper canine (total accuracy = 82%), this model did not yield significant results, rendering it unsuitable for use with these teeth. 

**Table 3:** Outcomes of the backward stepwise method based on the optimal predictors (1b, 7a, and 7c) for the lower canine root. The model was significant (Chi-square, p<0.001).

Predictor	Estimate	Standard Error	p-value	Odds ratio
1b	2.781	1.349	0.0392	16.137
7a	2.094	1.067	0.0480	8.121
7c	2.020	0.711	0.0050	7.540
Intercept	-31.690	7.374	<0.0001	0.000

1b, mesiodistal cervical diameter; 7a, axial volume at the cervical level; 7c, coronal volume at the longitudinal axis of the cervical two-thirds of the root.

When the upper and lower canines were analyzed together, the optimal model incorporated measurement 1b of the upper canine and measurements 1b and 1c of the lower canine. This model showed an overall accuracy of 89% (female = 92%, male = 86%), with a specificity of 92%, and a sensitivity of 86%. The validation accuracy of the formula derived from this model, using these measurements, exhibited moderate agreement (Kappa = 0.6, p < 0.0001) and an overall accuracy of 81% (female = 90 %, male = 70%).

The model with accuracy equal to or greater than 80% for each sex, which could be used to analyze compromised root length, was based on the lower canine measurements 1b and 7a (Table 4), achieving an overall accuracy of 87% in the evaluation sample. The formula (sex = -24.34 + (2.72 * 1b) + (2.81 * 7a) generated by this model demonstrated substantial agreement (Kappa = 0.7, p < 0.0001), with an accuracy of 85% (female = 90%, male = 80%). Table 5 shows the best models that, in the evaluation sample, reached an accuracy above 80%. Table 6 presents the best models for teeth with compromised tooth structure that, in the evaluation sample, showed accuracy above 80%.

**Table 4:** Outcomes of the Backward Stepwise method considering measurements 1b and 7a for the lower canine with compromised root length. The model was significant (Chi-square, p<0.001).

Predictor	Estimate	Standard Error	p-value	Odds ratio
1b	2.72	1.183	0.021	15.2
7a	2.81	0.95	0.003	16.6
Intercept	-24,34	5.491	<0.001	2.68-11

1b, mesiodistal cervical diameter; 7a axial volume at the cervical level.

**Table 5:** Best models in the evaluation sample with accuracy above 80%.

Model	Accuracy in the evaluation sample	Accuracy in the validation sample
UC 1b, 1c, 3	88%	63%
LC 1b, 7a, 7c	89%	85% (F=85%, M=85%)
UC 1b + LC, 1b, 1c	89%	81% (F=90%, M= 70%)

UC, Upper canine; LC, lower canine; 1b, mesiodistal cervical diameter; 1c, root length; 3, volume of the root; 7a, axial volume at the cervical level; 7c, coronal volume at the longitudinal axis of the cervical two-thirds of the root. The underlined values are the only ones where both overall and sex-specific accuracies meet or exceed 80%.

**Table 6:** The best models to be applied to compromised root structure.

Model	Compromised tooth structure	Accuracy in the evaluation sample	Accuracy in the validation sample
UC 7a	Root length	82%	78%
LC 1b, 7a	Root length	87%	85% (F=90% , M=80%)
UC 1b + LC 7a	Root length	88%	80% (85%, M= 75%)
UC 1b, 1c	Buccal cervical margin	81%	63%
LC 1b, 1c	Buccal cervical margin	84%	83% (F=90% e M= 75%)
UC 1b + LC 1b	Buccal cervical margin	82%	75%

UC, Upper canine; LC, lower canine; 1b, mesiodistal cervical diameter; 1c, Root length; 7a axial volume at the cervical level; 7c, coronal volume at the longitudinal axis of the cervical two-thirds of the root. The value underlined indicates the model in which the overall accuracy and the accuracy for each sex are equal to or higher than 80%.

## Discussion

Given that all assessed teeth in the present study exhibited sexual dimorphism, the null hypothesis was rejected. All measurements demonstrated that male roots are larger than female roots, consistent with previous studies ^4^ ^,^ ^9^ ^,^ ^10^ ^,^ ^11^ ^,^ ^14^ ^,^ ^17^. Alvesalo ^23^ states that the Y chromosome is responsible for dentinogenesis and amelogenesis, while the X chromosome primarily influences enamel formation. This genetic differentiation accounts for the larger dental structures observed in males. Despite the greater size of male dental structures, most studies, including the present one, report higher accuracy in sex estimation for females ^4^ ^,^ ^9^ ^,^ ^11^ ^,^ ^17^, potentially due to the lower variability in female tooth size. However, some studies have observed the opposite trend ^10^ ^,^ ^14^. It is essential to note that the female sample in the study by Adams and Pilloud ^10^ was substantially smaller.

When comparing the sex estimation accuracy obtained through volumetric analysis of the entire root with the accuracy reported by Manhaes-Caldas et al. ^6^, which evaluated crown volume (upper canine, 74.4%, lower canine, 79.5%), it was observed that the root (82% for both canines) exhibited superior accuracy in sex estimation. This finding corroborates earlier research indicating that the root portion is more reliable for sex estimation compared to the tooth crown. ^11^ ^,^ ^12^.

To expedite sex estimation, particularly in cases involving numerous victims, single-rooted teeth were selected for this study. Previous studies evaluating the Brazilian population ^6^ indicated that upper central incisors exhibit less sexual dimorphism than canines. While the upper central incisor, upper and lower canines are frequently identified as single-rooted teeth with pronounced sexual dimorphism, when analyzing dental crowns, two studies analyzing the root portion and multiple teeth, including upper central incisors, suggested that upper lateral incisors exhibit greater dimorphism ^13^ ^,^ ^14^. Consequently, upper lateral incisors and upper and lower canines were selected for analysis in this study. Although canines consistently exhibit significant dimorphism when analyzed separately, some studies suggest that the upper canine is the most dimorphic tooth ^7^ ^,^ ^13^ ^,^ ^14^. In contrast, other research identifies the lower canines as the most dimorphic tooth ^6^ ^,^ ^10^. The present study aligns with the latter perspective. However, it is noteworthy that Zorba et al. ^13^ and Kazzazi et al. ^14^ have suggested that the upper lateral incisors exhibit even greater dimorphism than canines. In the present study, while the lateral incisor does exhibit sexual dimorphism, the formula derived from its measurements cannot be used for sex estimation, as its accuracy falls short of the 80% threshold and is lower than that of the models based on canines. Notably, achieving 80% accuracy is essential to meet the Mohan and Daubert ^24^ admissibility criteria for acceptance as forensic evidence in court. Nonetheless, it is essential to highlight that the degree of sexual dimorphism in tooth size varies between populations due to both genetic and environmental factors ^12^. Therefore, the application of metric analysis for sex estimation should be carried out in populations whose tooth size is similar to that of the population used to develop the methodology ^25^.

In the scientific literature, analyses have predominantly focused on linear measurements, particularly the buccolingual and mesiodistal dimensions of the cervical portion of the root ^9^ ^,^ ^10^ ^,^ ^11^ ^,^ ^12^, but also on the root length ^13^. Less frequently, surface area ^4^ and volume ^4^ ^,^ ^14^ ^,^ ^17^ have also been examined. In the present study, the linear measurements of the buccolingual and mesiodistal dimensions of the cervical region were both dimorphic and larger in males, corroborating findings from previous studies ^9^ ^,^ ^10^ ^,^ ^11^ ^,^ ^12^. However, depending on the population and tooth analyzed, either the buccolingual or mesiodistal dimension may exhibit greater dimorphism. Concerning root length, the present study aligns with existing literature ^13^, demonstrating that male roots are statistically larger than female roots. Nevertheless, root length proved less accurate in sex estimation than buccolingual and mesiodistal measurements across all evaluated teeth. Despite the observed sexual dimorphism in linear measurements, they did not meet the Mohan and Daubert admissibility criteria ^24^ when analyzed alone or in combinations. Therefore, in the studied population, linear measurements cannot be used as evidence in a court of law, as their accuracy falls below the 80% threshold ^24^.

In the present study, the evaluation of the surface area was dimorphic in all analyzed teeth. However, this finding partially corroborates those reported by Fernée et al. ^4^, who identified sexual dimorphism in the surface area of the upper lateral incisors but not in the upper and lower canines. ^17^. García-Campos et al. ^17^ evaluated the area of the basal surface of the dental crown, which corresponds in the present study to the surface area of the axial cross-section in the cervical portion. The accuracy reported in their study (87.8%) was close to that found in the present study (84%). The aforementioned authors observed that surface areas are significantly larger in males, a finding also supported by the present study. They suggested that this difference may be attributed more to size variations than to surface complexity, which aligns with the present study's findings. The accuracy of sex estimation using surface areas was equal to or slightly lower than that achieved with volume assessments. Regarding volume measurements, the present study showed that these measurements were more accurate for sex estimation than linear measurements, corroborating findings in the scientific literature ^9^ ^,^ ^10^.

Despite the presence of sexual dimorphism in all types of measurements and the fact that volume measurement alone can exceed 80% accuracy, it is widely agreed that combining measurements and teeth yields greater accuracy ^6^ ^,^ ^10^ ^,^ ^17^, a finding corroborated by the present study. For the upper canine, isolated measurements of mesiodistal diameter (1b), length (1c), and total volume ^3^ achieved accuracies of 80%, 66%, and 82%, respectively. When combined, these three measurements achieved an accuracy of 88% in the evaluation sample (predictive equation). For the lower canine, measurements of mesiodistal diameter (1b), axial cross-section volume (7a), and two-thirds coronal cross-section volume (7c) showed accuracies of 80%, 84%, and 75%, respectively. However, when these measurements were combined, the accuracy reached 89% in the evaluation sample. Therefore, the equations were only validated in the present study with combined measurements. Additionally, studies have demonstrated that the use of artificial intelligence has resulted in increased accuracy in sex estimation, representing a promising approach for this type of assessment ^8^ ^,^ ^15^.

The overall accuracy obtained in the evaluation sample, considering the best equations provided by logistic regression, was 89% for the lower canine, 88% for the upper canine, 89% when both teeth were evaluated together, and 71% for the upper lateral incisor. Adams and Pilloud ^10^, evaluating the Japanese population, achieved an overall accuracy of 85.4% with the logistic equation involving measurements of the upper canine, 82.4% for the lower canine, and 85% for both canines. In the study by Kazzazi and Kranioti ^14^, evaluating the Iranian population, the accuracy for the upper lateral incisor was 100%, for the upper canine was 97.1%, and for the lower canine was 90.6%, Garcovich et al. ^2^, working with a Spanish sample, obtained an overall accuracy of 76.2% for the upper and lower canines. Viciano et al. ^11^, evaluating the Italian population, obtained an accuracy of 88.6% for the upper canine. The equation considering both the upper lateral incisor and upper canine reached an accuracy of 88.9%.

The equations were tested and validated in a different sample population subgroup, resulting in a decrease in sex estimation accuracy across all evaluated equations. For instance, the overall accuracy of the upper canine equation dropped to 63%. The lower canine equation achieved an accuracy of 85%, while the combination of upper and lower canines yielded an accuracy of 81%. In the study by Garcovich et al. ^2^, the initial accuracy for the Spanish population was 76.2%, which decreased to 70% in the validation group. In the study by Kazzazi and Kranioti ^14^, the accuracy obtained in the evaluation sample was maintained after validation; however, it is relevant to note that this study had a total sample size of 52 patients, comprising 32 males and 20 females.

The strength of the present study lies in the use of novel logistic regression formulas based on newly proposed measurements, specifically cross-sectional volumes. Additionally, the study used a large sample size (140 patients, with 100 in the evaluation sample and 40 in the validation sample), featuring an equal distribution between male and female patients and perfect pairing between ages, known sex, and measurements performed on CBCT. This sample size facilitated achieving a test power of 95% for the logistic regression equations. Such studies hold practical significance as a highly accurate method can streamline the identification process by reducing the number of comparisons required between *antemortem and postmortem* information, specifically by limiting comparisons to data from deceased patients of the same sex.

A limitation of the present study is that, although the quality was deemed adequate for clinical purposes, some degree of motion artifacts was inevitable due to respiratory movement, as the images were acquired from living subjects. Such artifacts, which are absent in cadaveric imaging, can impair image quality and complicate structural measurements. Additionally, the study faced constraints due to the unavailability of dental records, which precluded verification of orthodontic treatment history and dental trauma. Despite this limitation, patients presenting with lingual orthodontic retainers on the lower anterior dentition and apical rounding-indicative of previous orthodontic intervention-were excluded from the study. Furthermore, to mitigate potential confounding from previous dental trauma, patients with periapical lesions, even if their teeth appeared sound, were also excluded.

While all equations for the upper and lower canines achieved accuracy higher than 80% in the evaluation sample, only those for the lower canine - considering the healthy root structure (sex=-31.69+(2.781*1b)+(2.094*7a)+(2.02*7c) and compromised root lengths (sex=-24.34+(2.72*1b)+(2.81*7a) - sustained accuracy above 80% for both sexes in the validation sample. Consequently, these equations are deemed viable as complementary tools for sex estimation.

## Conclusion

In conclusion, the root portions of the upper lateral incisor, upper canine, and lower canine exhibited sexual dimorphism. However, only the combined analysis of measurements from the lower canine achieved an accuracy of 80% or greater for both sexes in the validation sample. The formulas derived from these measurements are suitable for supplementary use in sex estimation within the studied population. Furthermore, it is recommended that the root portion be prioritized for sex estimation when more accurate methods are unavailable.
